# Soil salinization in agriculture: Mitigation and adaptation strategies combining nature-based solutions and bioengineering

**DOI:** 10.1016/j.isci.2024.108830

**Published:** 2024-01-12

**Authors:** Paolo Tarolli, Jian Luo, Edward Park, Gianni Barcaccia, Roberta Masin

**Affiliations:** 1 Department of Land, Environment, Agriculture and Forestry, University of Padova, Agripolis, Viale dell'Università 16, 35020 Legnaro (PD), Italy; 2 Inner Mongolia Key Laboratory of River and Lake Ecology, School of Ecology and Environment, Inner Mongolia University, Hohhot 010021, China; 3 Earth Observatory of Singapore (EOS), National Institute of Education (NIE) and Asian School of the Environment (ASE), Nanyang Technological University (NTU), Singapore, Singapore; 4 Department of Agronomy, Food, Natural Resources, Animals and Environment, University of Padova, Agripolis, Legnaro (PD), Italy

**Keywords:** Soil science, Agricultural science, Agricultural soil science

## Abstract

Soil salinization is among the most critical threats to agriculture and food security. Excess of salts adversely affects soil structure and fertility, plant growth, crop yield, and microorganisms. It is caused by natural processes, such as dry climates and low precipitations, high evaporation rate, poor waterlogging, and human factors, such as inappropriate irrigation practices, poor drainage systems, and excessive use of fertilizers. The growing extremization of climate with prolonged drought conditions is worsening the phenomenon. Nature-based solutions (NBS), combined with precision or conservation agriculture, represent a sustainable response, and offer benefits through revitalizing ecosystem services. This perspective explores NBS that can be adopted, along with their challenges and implementation limitations. We also argue that NBS could not be enough to combat hunger in the world’s most vulnerable regions and fully achieve the Sustainable Development Goal – Zero Hunger (SDG2). We therefore discuss their possible combination with salt-tolerant crops based on bioengineering.

## Graphical abstract

**Figure:**
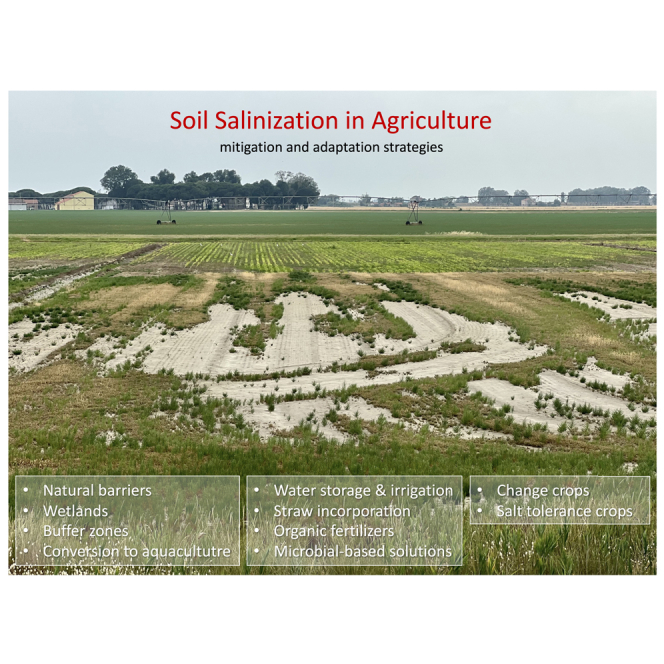

## Introduction

**Figure 1:**
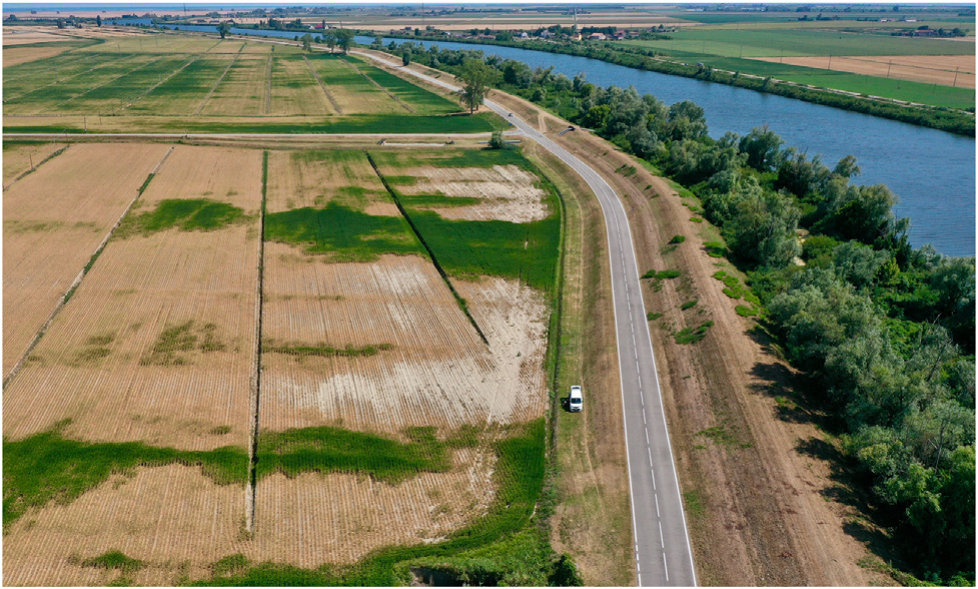
Saltwater intrusion effects (yellow and bare soil areas on the left side of the road) on soybeans in the Po River Delta (Italy) as seen from drone survey, 8 August 2022 (photo by S. Cucchiaro).

According to the UN 2030 Agenda for Sustainable Development, it is necessary to follow 17 Sustainable Development Goals (SDGs) to guarantee peace and prosperity for people and the planet. The purpose of SDG2 is to end hunger and ensure sufficient food for all people, particularly the poor. Sustainable food production systems need to be guaranteed through sustainable and resilient agricultural practices that increase productivity, maintain ecosystems, strengthen capacity for adaptation to climate change, and progressively improve land and soil quality. Unfortunately, the SGD2 target is at risk since climate change threatens global agriculture.^1^ At present, more than 8 billion people live on Earth. About 40% of the global population is located within 100 km from the coast, with over 600 million people at elevations less than 10 m asl.^2^ About 30% live in drylands where, according to UN-Habitat, the population growth rate was faster than in any other ecological zone. Important socioeconomic activities are located along coastal areas, and unfortunately, also a significant number of poor people. Global warming is threatening these regions by extremizing the hydrological cycle with an acceleration of the evapotranspiration and rainfall rate, megadroughts, flash droughts, and sea level rise. One of the effects, worsened by not optimal water resources management and excessive use of fertilizers, is the salinization of soils. This phenomenon is a rising concern since it could negatively impact agriculture and food security.^3^ Soil salinization represents a significant challenge for agricultural deltas worldwide.^4^^,^^5^ This includes key deltas in the Mekong (Vietnam) and Po (Italy) rivers and various regions across Africa and America. Here, sea level rise combined with saltwater intrusion has exacerbated the problem of soil salinization. In Southeast Asia, the Mekong Delta, a prominent rice production hub, faces an existential threat from saline intrusion.^6^^,^^7^ In 2020, this region saw an unprecedented peak in salinity levels, which penetrated more than 110 km inland and affected over half a million hectares of arable land.^8^ It damaged approximately 30,000 ha of paddy rice in Ca Mau province and about 20,000 ha of vegetables in Ben Tre province, threatening the livelihoods of millions dependent on the region’s agriculture. Likewise, in Europe, the Po River Delta in Italy is also grappling with this issue (Figure 1). Sea level rise, combined with subsidence (caused between the 1950s and 60s by extractions of methane from the subsoil), low river discharges and groundwater extraction, have led to increasing soil salinity, negatively impacting the delta’s unique agroecosystems.^9^^,^^10^^,^^11^^,^^12^

**Figure 2:**
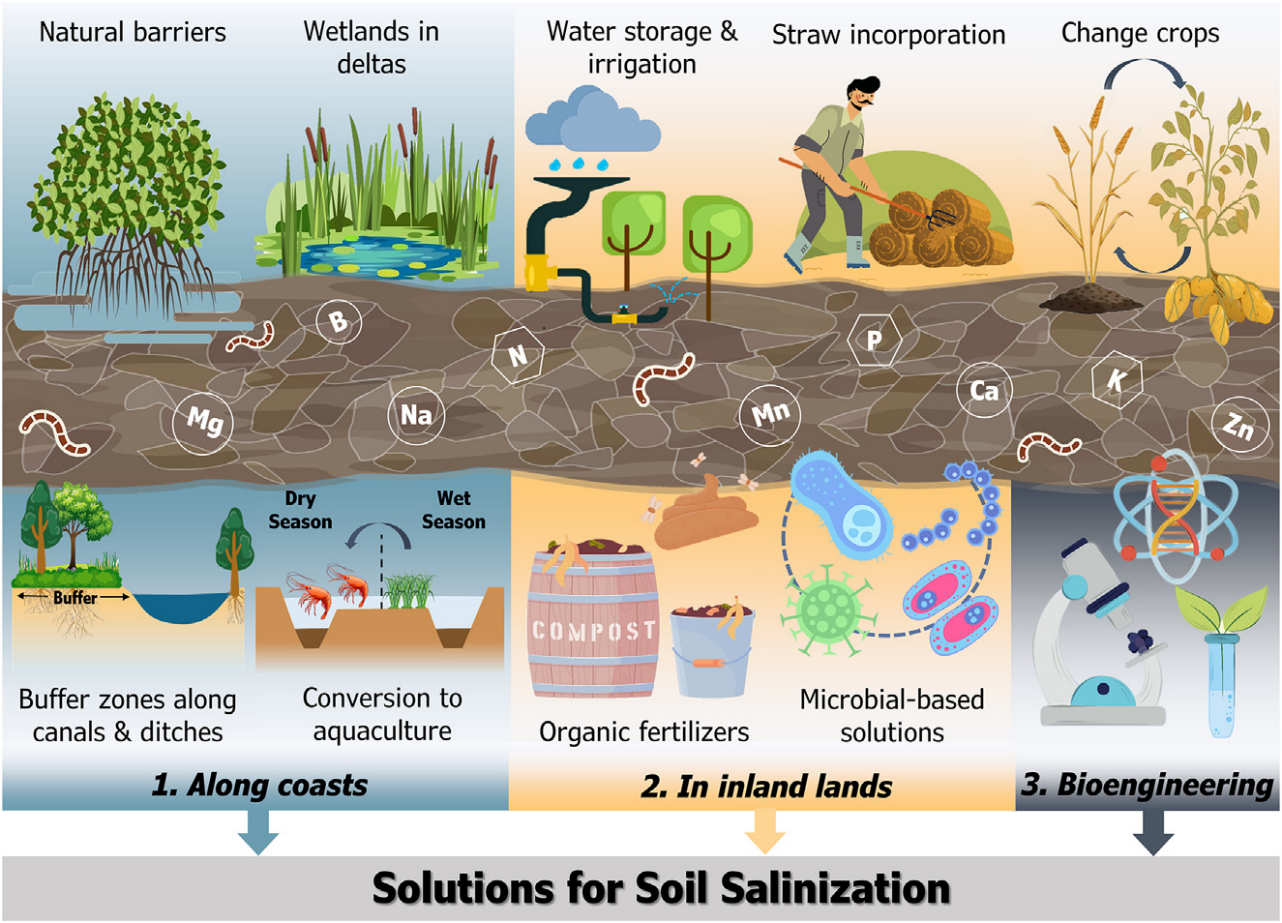
Conceptual framework illustrating sustainable solutions discussed in this paper, to mitigate soil salinization in agriculture

In Africa, coastal deltas from Egypt to Senegal face similar challenges. The Nile Delta, for instance, suffers from a complex interplay of sea level rise, reduced sediment flow, and anthropogenic activities that exacerbate saline intrusion, affecting as much as 35% of cultivated land.^13^^,^^14^ Lastly, North America’s Mississippi Delta is also afflicted by soil salinization. Intensive farming practices, rising sea levels, and periodic hurricanes have amplified saltwater intrusion, impairing soil fertility and crop productivity.^15^^,^^16^ These examples from various deltas around the globe highlight the pressing issue of salinity intrusion disasters and their severe consequences for agriculture in delta regions. The soil salinization phenomenon is widely spread also in inland arid regions.^17^ Inland deserts and arid lands cover vast expanses of the Earth’s surface, comprising diverse ecosystems such as deserts, steppes, and drylands. These regions are often fragile and highly sensitive to environmental disturbances, including changes in water availability and quality.^18^ These areas, characterized by low rainfall and high evaporation rates, are particularly susceptible to salinization due to limited freshwater resources and high soil salt concentrations.^19^ The causes of salinization in inland deserts and arid lands are multifaceted. Natural processes, such as weathering rocks and minerals, release salts into the soil and water.^20^ In Western Australia, for instance, rock weathering and deposition over thousands of years has resulted in salinity accumulation of approximately 100 and 15,000 tonnes/ha.^21^^,^^22^ However, human activities play a significant role in accelerating salinization processes. Improper irrigation practices, excessive groundwater extraction, and inadequate drainage systems can accumulate salts in the soil profile, gradually rendering the land unsuitable for cultivation.^23^^,^^24^^,^^25^ Soil salinization jeopardizes the productivity of agricultural lands, degrading soil fertility and threatening the survival of plant and animal species adapted to arid conditions.^26^ Addressing the challenges posed by soil salinization requires a multi-faceted approach. To mitigate such phenomenon, it is necessary to adopt sustainable water management practices, promoting efficient irrigation techniques (e.g., drop and sub-irrigation) combined with rainwater harvesting facilities. However, only with the adoption of NBS it is possible to solve the issue while preserving ecosystem services. The purpose of this perspective is to explore the capability of NBS to mitigate soil salinization in agriculture through a combination of sustainable solutions that can be adopted from coastal areas to inland arid lands. We also debated the possible benefit of bioengineering advances in selecting and creating salt-tolerant crops. Indeed, under the urgent need to combat hunger, given the actual acceleration of climate change, NBS could not be enough to act in large areas and support millions of people. Therefore, we propose combining salt-tolerant crop varieties with NBS to optimize the benefits in the world’s most vulnerable regions. For each solution described, we addressed the challenges and limitations of their implementations. A conceptual framework of the mitigation solutions described in the work is summarized in Figure 2.

## Saltwater intrusion along coasts

### Natural barriers

Nature-based solutions (NBS) have emerged as a promising approach to tackle the challenges posed by sea level rise and soil salinization. NBS, defined as “actions to protect, sustainably manage, and restore natural or modified ecosystems that address societal challenges effectively and adaptively, simultaneously providing human well-being and biodiversity benefits”,^27^ utilize natural barriers, such as mangroves, saltmarshes, and seagrass meadows, to provide sustainable and effective mitigation strategies.^27^^,^^28^^,^^29^ In the Mekong Delta, NBS has been increasingly adopted in recent years. Historically, the region was characterized by vast mangrove forests that serve as natural buffers against saline intrusion, absorbing the impact of higher tides and preventing the penetration of saltwater inland.^30^ They also played a crucial role in enhancing soil stability and fertility through sediment capture and organic matter accumulation,^31^ promoting agricultural resilience. However, extensive deforestation in recent decades has severely diminished the Mekong’s mangrove coverage, exacerbating salinity problems.^32^^,^^33^ In the Ben Tre Province of the delta, for instance, 50% of mangrove forest have been cleared between 1998 and 2015, severely reducing rice yields.^34^ Instead, locals are shifting to more salt-tolerant crop production such as sugarcane, coconut, and orchard.^35^ Recognizing mangroves’ importance led to many mangrove restoration projects by local communities and international organizations, effectively promoting a 11,184 ha growth in mangrove forest area in the Vietnamese Mekong Delta between 2015 and 2020.^36^ By reintroducing mangrove forests and integrating them into the agricultural landscape, these initiatives aim to restore the delta’s natural defenses against salinity and secure the region’s agricultural productivity. Restoring and maintaining these natural deltaic ecosystems serve as a sustainable, nature-based shield against the encroaching threats of soil salinization and sea level rise. In temperate climates like the Netherlands, marsh restoration projects are also increasingly adopted to tackle soil salinity and flood mitigation measures.^37^ While NBS shows promise, their implementation is not without a challenge. These include land tenure issues, insufficient local knowledge, and upfront costs that may deter investments.^33^ Moreover, the effectiveness of these solutions can be geographically contingent. For example, mangrove restoration may be highly effective in tropical regions like the Mekong Delta, but less in the colder climates where these plants may struggle to thrive. Similarly, salt marshes are more suitable for areas with specific tidal ranges and sediment types.^28^ Therefore, understanding regional specificities is essential for successfully adopting NBS for combating soil salinization.

### Wetlands in deltas

Inland delta wetland restoration presents another compelling NBS that can help tackle salinity intrusion in deltas.^31^^,^^38^^,^^39^ This approach leverages the inherent ability of wetlands to hold and filter water, recharge groundwater resources, and resist the infiltration of saline water from the coast. Functioning as natural reservoirs, wetlands collect and store freshwater during high-precipitation periods, releasing it gradually over time.^40^^,^^41^ This consistent freshwater release replenishes groundwater systems and aquifers, elevating water tables and creating a counter-pressure against saline water intrusion. Thus, this natural hydrological buffering system can be amplified by restoring and conserving wetlands in the inland delta regions. In the context of the Mekong Delta, where aquifer over-extraction has accelerated saline intrusion,^42^ wetland restoration such as that in Can Gio Biosphere Reserve, has played a pivotal role in rebalancing the fresh-saline water interface. This has enhanced water security and improved soil health, biodiversity, and carbon sequestration.^43^^,^^44^ Moreover, wetland restoration supports socio-economic development in delta regions, providing benefits such as flood mitigation, improved fisheries, and eco-tourism opportunities.^45^^,^^46^ Thus, from an NBS perspective, restoring and maintaining wetlands is a holistic approach to addressing the saline intrusion issue while delivering multiple co-benefits. While wetland restoration offers numerous benefits, its implementation also faces challenges such as land use conflicts, regulatory hurdles, and the need for interdisciplinary expertise for effective ecosystem management.^45^ Funding for large-scale restoration projects can also be a significant limiting factor. Geographically, wetland restoration is more effective in regions with existing or historical wetland ecosystems. However, in arid areas or those with a history of extensive land reclamation, the efficacy of this approach may be limited. Hence, inherent physiographical factors like climate, geology, and land use history must be factored in for an efficient NBS.

### Buffer zones along canals and ditches

The adoption of buffer zones along canals and ditches could represent an effective strategy to mitigate the potential intrusion of saltwater. They can be a barrier between freshwater aquifers and saline water bodies.^47^ The roots of the plants prevent erosion and maintain the integrity of the buffer zone. Additionally, the plants can absorb excess water, reducing the hydraulic pressure and minimizing saltwater intrusion. Native salt-tolerant plant species could be optimal for these buffer zones, as they are adapted to thrive in saline environments. Therefore, they need to be adapted to climate zones and be site-specific. Buffer zones not only safeguard freshwater resources but also preserve fragile coastal ecosystems. Indeed, these zones provide habitats for various plant and animal species, contribute to biodiversity conservation, and enhance overall ecosystem resilience. Challenges in their implementations are several. First, they reduce productive farming areas and potentially affect farmers’ income. This land-use conflict can limit the widespread adoption of buffer zones. Another limitation is the lack of uniform regulations and guidelines. Buffer zone requirements vary from region to region, making it confusing and difficult for farmers to comply. The lack of standardized practices can lead to inconsistent implementation and enforcement. Maintaining buffer zones can also be resource intensive. Farmers must invest time, labor, and resources in managing these areas. Overcoming these challenges, through subsidies and clear ad-hoc guidelines, is essential for maximizing the well-known and widely acknowledged benefits of buffer zones in agriculture.

### Conversion to aquaculture

Farmers in delta regions worldwide are consistently developing adaptive strategies to mitigate the negative impacts of saline intrusion on their livelihoods.^48^^,^^49^ Particularly noteworthy is the innovative approach farmers adopt in the Mekong Delta.^7^^,^^50^^,^^51^ In the face of escalating soil salinization, farmers in the Mekong have introduced rice-prawn rotational farming, a system that not only adapts to but also capitalizes on saline conditions.^7^^,^^52^ This agroecological model involves cultivating rice during the rainy season when freshwater availability is high and switching to prawn farming in the dry season when salinity levels rise. The benefits of this approach are multifaceted and have supported the livelihoods of millions of people.^53^ The rice crops, vital for food security, benefit from the nutrient-rich soil left by the prawns, reducing the need for synthetic fertilizers. On the other hand, prawn farming offers a profitable venture due to the high market demand, providing an alternative income stream to counterbalance potential yield reductions in rice crops due to salinity. This rice-prawn farming system exemplifies how agricultural practices can adapt to environmental challenges. It represents a resilient response to saline water intrusion that aligns with the principles of NBS – harnessing natural processes to create sustainable and adaptive agricultural systems in an era of changing climate and rising sea levels. The challenges, however, exist in its adoption despite the great promise shown by the rice-prawn rotational farming system. One key issue is the initial investment required for infrastructure modifications to support rice and prawn farming.^52^ There is also a lack of motivation to master dual farming practices, and farmers may require technical training and support.^7^ Water quality management is another hurdle, as improper practices can lead to contamination that could jeopardize rice and prawn yields. Moreover, market fluctuations in the prawn price may also introduce financial instability for farmers reliant on this dual-cropping system.

## Saltwater in inland arid lands

### Straw incorporation

Strategies involving the incorporation of straw have demonstrated their effectiveness in mitigating soil salinization. A significant advantage of straw incorporation lies in its capacity to augment the organic matter content within the soil. Rich in carbon, straw is a valuable food source for soil microorganisms. These microorganisms play a crucial role in decomposing organic matter, releasing vital nutrients, and enhancing the soil structure.^54^^,^^55^ The heightened organic matter content contributes to improved soil water-holding capacity, thereby decreasing the concentration of soluble salts and preventing their accumulation in the root zone.^56^ Moreover, straw incorporation fosters the development of stable soil aggregates, creating pore spaces that facilitate air and water movement.^57^ This process aids in leaching excessive salts deeper into the soil profile, away from the root zone. Additionally, incorporating straw enhances soil structure, facilitating better plant root penetration and nutrient uptake.^58^^,^^59^ Various techniques can be employed for straw incorporation, tailored to the specific requirements of the soil and crops. These techniques encompass ploughing, disking, or harrowing the straw into the soil and adopting conservation tillage practices that leave the straw on the soil surface. Their implementation faces, however, multifaceted challenges. Ensuring appropriate incorporation depth demands technical expertise. Inconsistent straw quality and quantity could impact effectiveness. Straw incorporation could significantly increase soil methane emissions,^60^ and slow straw decomposition in fields, coupled with excessive residue, could hinder crop sowing quality, germination, and early growth.^61^

### Microbial-based solutions

Cohabitation with halotolerant microorganisms, particularly bacteria, enhances the ability of plants to withstand salinity stress. The increased plant growth induced by these bacteria is a cumulative outcome of diverse mechanisms at both physiological and molecular levels, which are concurrently activated.^62^ These microorganisms promote plant growth, improve stress tolerance, and enhance nutrient absorption.^63^ Consequently, microbial inoculations present promising NBS for addressing salinity-related challenges in agriculture. However, it is crucial to accurately select suitable and efficient bioinoculants.^64^ It should be noted that the efficiency of salt-tolerant plant growth-promoting rhizobacteria may vary depending on the climate in different ecological zones.^55^ In field conditions, certain bacterial strains may exhibit an inability to successfully colonize the roots, resulting in a diminished capacity to promote plant growth compared to laboratory-controlled conditions. To avoid failure, the crop variety, and the specific salt-tolerant plant growth-promoting rhizobacteria strain employed must be carefully considered. In-depth laboratory and field studies are necessary to identify suitable environmental-friendly bioinoculants that facilitate crop growth under saline conditions. Regardless, there is increasing empirical evidence that, by implementing inoculations on a large scale, agricultural sustainability in the saline environment could be advanced.^64^ It must be observed that, while promising, this practice is not without criticisms and is attracting growing attention as the market of soil microbial inoculants expands. Introducing microbial inoculants into new ecosystems raises concerns, as the release of alien species poses a potential risk of compromising ecological integrity and endangering indigenous communities vulnerable to introduced species. The long-term impact of introducing microorganisms into new ecosystems thus requires comprehensive ecological evaluations before widely embracing this practice.

### Organic fertilizers

Using organic fertilizers offers an effective strategy for preserving soil organic carbon (SOC) levels and preventing soil salinization. Derived from natural sources like animal manure, compost, or cover crops, organic fertilizers deliver numerous benefits to soil health and fertility.^65^ By introducing organic matter into the soil, these fertilizers contribute to SOC enrichment. This organic matter serves as a carbon source that stimulates the activity of soil microorganisms, leading to the decomposition of organic materials. Consequently, this process releases essential nutrients into the soil, supporting plant growth while increasing SOC levels. By maintaining or augmenting SOC, organic fertilizers enhance soil structure and bolster water-holding capacity,^66^ effectively reducing the risk of soil salinization. Furthermore, organic fertilizers are vital in enhancing nutrient cycling and availability in the soil. The organic matter in these fertilizers acts as a reservoir of nutrients, slowly releasing them over time, thus reducing the likelihood of nutrient leaching and runoff.^67^ This efficient nutrient management helps maintain a balanced soil ecosystem and prevent the accumulation of salts, a major cause of soil salinization. Having said that ensuring a consistent and reliable supply of organic materials can be difficult due to factors such as availability, transportation, and cost. Second, determining the optimal application rates and timing of organic fertilizers demands a thorough understanding of soil characteristics and crop requirements. Third, organic fertilizers’ nutrient content and decomposition rates can vary significantly, affecting their effectiveness in addressing salinity.^68^ Moreover, the impact of organic fertilizers on soil structure, nutrient retention, and microbial activity can differ based on soil types and environmental conditions.^65^

### Change crops

It is imperative to adopt effective crop strategies to maintain agricultural productivity and prevent crop overstress caused by salty conditions. One essential approach is the careful selection of salt-tolerant crops. These crops possess genetic characteristics that allow them to thrive even in environments with elevated salinity levels.^69^ Another beneficial practice is implementing crop rotation techniques. By alternating salt-sensitive crops with salt-tolerant varieties, the cycle of salt accumulation can be disrupted, alleviating plant stress and preserving soil fertility.^70^ Despite residual salinity and soil deterioration due to salinity that may affect both spring/summer crops and winter crops, salinization is often observed as a seasonal phenomenon in specific regions and critical salinity levels are reached primarily in summer.^71^ In such areas, opting for winter crops, particularly those with higher salinity tolerance, i.e., barley, can be another advantageous option. Incorporating cover crops into crop rotation to be used as mulch for the main crop might be another promising practice. Another issue to consider is the intricacies of potential shifts in pests and diseases linked to different crops adds to the challenges. Therefore, before acting is better to have a comprehensive agronomic analysis of potential implications and a suitable risk management analysis of potential new threats. A completely different alternative is the implementation of agroforestry systems, which are mixed systems that combine herbaceous and woody species for both productive (with a preference for fruit trees) and protective purposes. Agroforestry can serve as a more effective solution for managing salinity soil, as it has the potential to enhance soil and environmental quality while increasing the variability of farm production. Trees integrated into the farming system can provide a source of food, fodder, timber, and fuel, and contribute to resource conservation through afforestation, thereby promoting environmental sustainability.^23^ None of these strategies can be considered conclusive alone, but the combination and synergy of cited approaches can contribute to maintaining satisfactory profitability for farmers in a saline environment. Adapting crops for soil salinization mitigation varies significantly across different climate zones due to geographical and environmental factors. Overall, the action is to promote salt-tolerant varieties that can be easily adapted to the climate condition of the region. To provide a few examples, farmers can consider transitioning to salt-tolerant tropical crops in tropical regions characterized by high temperatures and heavy rainfall, such as rice varieties specifically bred for salinity tolerance. Mediterranean climates, known for their mild, wet winters and hot, dry summers, can increase salinity during the dry season. In such regions, farmers can adapt by introducing salt-tolerant crops native to the Mediterranean, such as certain olive tree varieties or explore other solutions where olive tree is inappropriate. Farmers can prioritize winter salt-tolerant crops like winter wheat and rye in cold climates.

### Water storage and irrigation

Water management is crucial in mitigating the effects of soil salinization.^72^ Rainwater harvesting facilities need to be promoted and optimally designed to be efficient.^73^ These could be micro or macro storages: micro serves as the optimal solution to guarantee farm-scale freshwater for emergency irrigation; macro is more aligned with the district-scale needs (e.g., freshwater for public supply). They can help reduce groundwater exploitation along coastal areas and constitute a natural refuge for birds and amphibians, improving biodiversity. Rainwater harvesting infrastructures must be related to advanced irrigation systems. Implementing more efficient irrigation practices by optimizing watering schedules and reducing water consumption is important. Even using moderately salted water for irrigation can increase soil salt content. Therefore, considering the detrimental impact of over-optimal irrigation on exacerbating salinization in saline agriculture, a shift toward precision irrigation is necessary, employing irrigation models and water-demand mapping. Among irrigation methods, micro-irrigation offers various advantages. The leaves are not exposed to saline water, which can harm the plant tissues; moreover, due to its frequent application, micro-irrigation enables continuous leaching of the soil volume from which the plant extracts water. The wetting pattern around emitters in micro-irrigation leads to a higher leaching fraction and lower salinity levels compared to other irrigation systems when the same amount of water is applied.^74^ This wetting desalination zone can gradually extend deeper into the soil with continuous irrigation. Drip irrigation, if optimally applied with a pre-designed appropriate irrigation volume can offer multiple benefits: water saving but also salt suppression.^75^^,^^76^ Subsurface drip irrigation is even more efficient since it reduces soil evaporation and facilitates better moisture distribution than conventional drip irrigation.^3^ Unlike traditional surface irrigation, sub-irrigation delivers water directly to the root zone of plants, minimizing evaporation and wastage and, at the same time, preventing saltwater from being absorbed by the roots, therefore, it is an optimal practice for arid lands. Limitations in adopting such strategies are related to costs, which can limit large-scale implementation, especially for low-income countries. Concerning rainwater harvesting solutions, the size may not be sufficient to meet the water needs of large-scale commercial farming operations, or on the other hand, the climate is so dry that the rain collected is not enough to support agriculture. In such cases, supplementary water sources may still be required; therefore, more structured water reservoirs and groundwater use must be implemented. However, this latter solution has a price in terms of environmental impact; if groundwater exploitation reaches unsustainable levels, a collapse of soil ecosystems and entire communities without freshwater may be possible.

## Breeding for salt tolerance in crop plants

Salinity is one of the most important environmental stresses affecting plant growth and development, reducing crop yield. Plants are known to promote adaptation mechanisms to tolerate saline conditions by activating or modulating specific genes that can regulate osmotic and oxidative stresses induced by salinity.^77^ Breeding crops and ornamental plants for salinity tolerance is challenging but possible, and significant improvements have been achieved in the last years by selecting salt-tolerant genotypes in model species.^78^^,^^79^

Nowadays, in the era of structural and functional genomics, the genetic factors, as well as the physiological and biochemical mechanisms underlying salinity tolerance, have been widely investigated and unveiled for the major crop plants, with potential applications in molecular breeding programs.^77^^,^^78^^,^^79^ Several quantitative trait loci (QTLs) related to salinity stress have been mapped by genome-wide analysis studies as well as candidate genes encoding for transcription factors and specific proteins induced by salinity stress have been identified in crop plants (reviewed by Afzal et al.^77^). This information makes now possible to combine conventional and molecular breeding approaches. In the past few decades, marker-assisted selection has deeply transformed plant breeding by giving breeders the foreknowledge of critical traits during the seedling stage, allowing them to make phenotypic predictions early on and with greater precision and accuracy.^80^ However, one of the major challenges in both conventional and molecular breeding is represented by the linkage drag, which depends on the unwanted transfer of undesirable linked genes from wild species to cultivated varieties. To overcome this drawback, genome editing technology has more recently provided a solution by making possible and reproducible the specific modification of desired traits using gene knockout or gene replacement strategies: genome editing not only revolutionized plant biology and, more in general, life sciences^81^ but it has provided the means to solve challenges related to crop yield and food security, nutrient content and quality, resistance or tolerance to diseases and adaptation to environmental constraints.^82^ This technology has provided new opportunities for easily improving plant varieties for desirable traits controlled by single genes or polygenes, including some that are agronomically important and specifically related to resilience to climatic changes and environmental stresses, such as those responsible for drought and salinity tolerance.^77^ High-level tolerance to salinity includes modifying several genes, including those associated with morphological and anatomical variations in plants and involved in the transportation and partition of KCl, NaCl, and Cl at genetic and epigenetic stages. Conventional breeding strategies mediated by intervarietal crosses combined with several backcrosses can be useful to pyramid Mendelian genes and polygenes at QTLs. In addition, new genome or gene editing platforms provide disruptive options to boost salt tolerance using CRISPR/Cas-based applications. This technology is expected to be highly useful for breeding new varieties throughout targeted and accelerated crop improvement, specifically focusing on resilient traits.

Agriculture urgently demands to develop new plant materials, resistant or tolerant to climate changes, adaptable to marginal lands, and suitable to low inputs and environmental stresses. The new breeding techniques, including gene and genome editing platforms, combined with precision agriculture under sustainable farming systems, answer this specific need.^83^ In our view, the potential use of new biotechnological approaches for either loss-of-function and gain-of-function applications, such as cisgenesis (for conspecific gene transfer or introgression) and genome editing (for endogenous gene knockout or silencing and gene editing or replacement) will assume strong relevance in plant breeding programs dealing with tolerance to abiotic stresses, as these methods would allow direct intervention at the genomic level in any variety/cultivar without changing its genetic background.^84^ We are confident that these applications of genomics combined with platforms of phenomics represent the right answer for the future of agriculture as it can provide a key contribution to the development of new plant genotypes and molecular assays for the main crop species to implement and predict the agronomic value-added of plant varieties expressing resilient traits and phenotypes, including those for salt tolerance.

In conclusion, combining rapidly advancing new genomic technologies with breeding and farming methodological advancements will greatly increase crop yields, food quality, and the overall sustainability of agricultural systems.^83^ We are confident that next-generation crop plant varieties expressing salt tolerance will play a crucial role in making productive processes more actionable with specific environmental constraints and more sustainable from economic and social points of view. There is no doubt that genetic improvement using conventional plant breeding methods has been a central pillar of enhanced agricultural productivity for more than a century. However, genetically engineered or edited varieties that manifest resistance or tolerance to abiotic stresses, including salinity, are now increasingly important worldwide and represent an irreplaceable source of improved plant materials that may complement advanced management plans of precision or conservation agriculture. Despite the advantages associated with these varieties in terms of field adaptation and crop yields, by simplifying the systems agronomy approach and improving the growth efficiency of plants, there are also challenges for successful implementation and systematic utilization. One of the biggest challenges for developing genetically engineered or edited varieties is the need to identify distinct germplasm resources and transfer or edit genetic variants that confer high salt tolerance in the main crop plant species. Finally, we would also like to emphasize the importance of agricultural community engagement and commitment, modern regulatory policy, strong partnership or agreement among research centers and seed companies, along with education and training initiatives dedicated to farmers for achieving long-term success of breeding programs aimed at the improvement of salt tolerance in crop plant varieties and mitigation of soil salinization effects in agriculture.

## Concluding remarks

In this perspective, two approaches to mitigate the growing phenomena of soil salinization in agriculture are proposed: NBSs and breeding for salt tolerance in crop plants. NBSs have the advantage of revitalizing ecosystem services and contributing to biodiversity conservation; however, they are not always feasible since climate conditions could be a limited factor for the effectiveness of some species. Bioengineering in selecting and creating salt-tolerant crops could be a valid alternative, especially for large-scale agricultural systems where preserving one crop is essential to guarantee a specific food need or some crops hold significant cultural and traditional importance for people living in the area. The indication is to implement both approaches to fully achieve SDG2 while guaranteeing environmental sustainability.
